# PROTOCOL: Police stops to reduce crime: A systematic review

**DOI:** 10.1002/cl2.1166

**Published:** 2021-05-06

**Authors:** David Weisburd, Kevin Petersen, Taryn Zastrow, Robert Davis, Lorraine Mazerolle, Elizabeth Eggins

**Affiliations:** ^1^ George Mason University Fairfax Virginia USA; ^2^ Hebrew University Jerusalem Israel; ^3^ George Mason University Fairfax Virginia USA; ^4^ National Police Foundation Arlington Virginia USA; ^5^ Department of Humanities and Social Sciences, School of Social Sciences University of Queensland St. Lucia Queensland Australia

## 1 BACKGROUND

### 1.1 The problem, condition, or issue

The use of pedestrian stops has been one of the most common yet controversial proactive strategies in modern policing (Weisburd & Majmundar, 2018). The pedestrian stop (also known as stop and frisk, *Terry* stops, street pops, stop and search, street stops, etc.) is often defined as the process by which “…officers stop, and potentially question and search, people in the communities they are patrolling” (Lachman et al., 2012, p. 1). These tactics have been staples in policing for generations, but they gained legitimacy with the landmark U.S. Supreme Court decision in *Terry v. Ohio* (1968)—which allows police officers discretion to conduct an investigatory stop of a person given reasonable suspicion that the person has committed a crime or is in the process of committing a crime, and discretion to frisk (or pat‐down) the person given reasonable suspicion that they are carrying a weapon (see Jones‐Brown et al., 2010).

Often termed “stop, question, and frisk (SQF)” (Rosenfeld & Fornango, 2014, p. 96), evidence suggests that many U.S. police departments began using pedestrian stops widely as a proactive policing strategy in the 1990s and early 2000s (Gelman et al., 2007; White & Fradella, 2016). In New York City alone, recorded SQFs increased from 160,851 in 2003 to 685,000 in 2011 (Weisburd et al., 2016), and similar increases have been noted in other U.S. cities such as Philadelphia and Los Angeles (Jones‐Brown et al., 2010; Saul, 2016). Police “stop and search” (McCandless et al., 2016, p. 2) powers have also been noted in the UK, where targeted pedestrian stops have been used as a strategy to reduce knife crime (Tiratelli et al., 2018), and in other European countries such as Bulgaria, Hungary, and Spain, often for the purpose of conducting identity checks related to criminal investigations (Miller et al., 2008). In this context, pedestrian stops have been used as primary components in a number of different proactive policing interventions, including crackdowns (Sherman, 1990), efforts to reduce illegal gun carrying (Koper & Mayo‐Wilson, 2006), directed patrol interventions (Ratcliffe et al., 2011), and hot spots policing interventions (Braga, Turchan, et al., 2019).

While advocates have considered pedestrian stops to be a contributing factor to decreasing levels of crime in American cities (Baker & Goldstein, 2012), critics have pointed to the low success rates (i.e., low proportions of stops that lead to arrest or weapon seizure) and racial disparity associated with these strategies as evidence that such tactics represent an illegal and unjust use of police power (Fagan & Davies, 2000; Gelman et al., 2007; Rosenfeld & Fornango, 2014). Racial and ethnic profiling has also been a concern on an international level, with researchers noting racially disparate stop rates in several European countries—without clear evidence that these strategies have produced meaningful crime reductions (McCandless et al., 2016; Miller et al., 2008; Tiratelli et al., 2018).

Despite such challenges, practitioners still view pedestrian stops as an important element of proactive crime prevention efforts (D'Onfrio, 2019; Terkel, 2013), making an understanding of their effects on crime and the community increasingly important. We propose a systematic review on the effects of pedestrian stops as a strategy for reducing crime, including examination of the degree of geographic focus of the programs examined, and possible moderators such as its linkage with other crime prevention approaches. In addition, this review seeks to examine the effects of pedestrian stops on the people and the communities (micro, meso, or macro) within which these strategies are employed.

### 1.2 The intervention

Pedestrian stops generally involve the police‐initiated stop of a person (or group of people) on the street for the purpose of investigation and/or questioning (Lachman et al., 2012). In most cases, the officer must have reasonable suspicion that a person is involved in criminal activity for a stop to occur, and based on the level of suspicion, a frisk or search of that person may be conducted. However, in certain contexts stops may be conducted without suspicion or the threshold for reasonable suspicion may vary. In the UK, the Criminal Justice and Public Order Act of 1994 permits suspicion‐less stops in high‐risk areas with approval from an authorizing officer (Lennon, 2013, 2015). Police officers in the UK and other European countries are also permitted to conduct suspicion‐less stops of people in authorized areas as a proactive counter‐terrorism measure (Lennon, 2013). Similarly, the U.S. Supreme Court has ruled that the amount of crime in a given area can be used as a factor in an officer's determination of reasonable suspicion (Gelman et al., 2007; *Illinois v. Warlow*). Thus, it is important to note that while pedestrian stops are often reactive in nature, in that they require prior indication of suspicious behavior or criminal activity, they may also be used proactively. In this regard, police stops are often employed as key elements of policing strategies that “…have as one of their goals the prevention or reduction of crime and disorder” (Weisburd & Majmundar, 2018, p. 1). Thus, pedestrian stops are used to both respond to observed or reported criminal behavior as well as to deter and prevent future criminal behavior.

Pedestrian stops may be employed as distinct policing interventions or used as components of larger interventions such as short‐term police crackdowns (Sherman, 1990), directed patrol presence (McGarrell et al., 2001; Ratcliffe et al., 2011), and hot spots policing (Weisburd et al., 2014). Additionally, while pedestrian stops have been primarily implemented as a tactic to reduce violent and/or weapon‐related crime (Koper & Mayo‐Wilson, 2006; Ratcliffe et al., 2011; Sherman et al., 1995), evidence has suggested that they have also been used to target other crime/disorder problems, for example, drug‐related crime (Geller & Fagan, 2010; Levine & Small, 2008). The current review will include any study that meets our methodological quality threshold (see later) in which different “regimes” of pedestrian stops are compared. This can be as a result of a policing intervention that employs pedestrian stops as a primary component, or as a result of controlled comparisons between areas that evidence varying levels of pedestrian stops, regardless of what (if any) specific crime/disorder outcome is being targeted. However, interventions employing pedestrian stops, but where such stops are not a primary programmatic intervention or the primary focus of the comparison, will be excluded.

### 1.3 How the intervention might work

It has often been argued that offenders weigh the potential costs and benefits associated with a criminal act, and as such, are deterred from crime in situations in which the potential costs outweigh the potential benefits (Beccaria, 1986; Bentham, 1988; Durlauf & Nagin, 2011; Nagin, 2013). Pedestrian stops may deter crime by increasing these perceived costs, and likewise the perceived certainty of apprehension among potential offenders (Lachman et al., 2012). In other words, people who have been personally stopped by the police may alter their behavior or avoid the area in which the stop occurred to mitigate their risk, while people who become vicariously aware of the pedestrian stop intervention may pre‐emptively do the same (Rosenfeld & Fornango, 2014). Furthermore, if pedestrian stops result in the seizure of weapons and other items that are often used to commit crime, they may reduce crime simply through the incapacitation achieved by preventing access to the tools needed to commit criminal acts (Sherman et al., 1995). It is also possible that pedestrian stop strategies deter crime merely through increasing police presence in high‐crime areas. In this context, the increase in deterrence is not necessarily related to the strategy itself, but rather to the increased presence of police in an area.

It is key in any policing program to examine the impacts of specific policing strategies on both the people targeted and the communities where they are applied. Advocates of pedestrian stops focus on the benefits of reduced crime in the community (D'Onfrio, 2019; Terkel, 2013). Critics of the approach argue that pedestrian stops may negatively affect community attitudes toward the police by increasing involuntary contact with community members in ways that are perceived as unfair and unlawful (Miller & D'Souza, 2015; Tyler et al., 2014). The negative effects of these stops may also stem from their disproportionate application to specific groups of people. For example, like other proactive crime prevention strategies that deploy police resources in high‐crime areas, pedestrian stops are often concentrated in disadvantaged minority communities (Fagan & Davies, 2000; Levine & Small, 2008). Given this concentration and the potential for officers to act on implicit biases that define racial minorities as safety threats (Epp et al., 2014; Holmes, 2000), there is concern that pedestrian stop strategies have disparate impacts on minority populations (Fagan & Davies, 2000; Ferrandino, 2015). For instance, research has suggested that in New York City, Blacks are over six times more likely to be stopped by police than Whites, and that the rate of success during these stops (operationalized as the rate of drug/weapon seizures or arrests) is often < 3% for seizures and < 7% for arrests (see Geller & Fagan, 2010; Gelman et al., 2007; Jones‐Brown et al., 2010). Thus, the vast majority of police stops appear to be conducted against disadvantaged populations that are neither committing an arrestable offense, carrying weapons, or carrying contraband.

As a result of these factors, police‐initiated stops may reduce feelings of police legitimacy among the people stopped or the communities in which they are found. In turn, there is evidence that pedestrian stops may lead to negative mental health effects on those stopped, such as anxiety and trauma (Geller et al., 2014)—because of the stress and fear engendered among populations such as young black males who are often more likely to be stopped in these programs. Pedestrian stops may also be conducted in a rough manner, potentially leading to the use‐of‐force that results in physical injury, or even death (see Miller et al., 2017), to the person stopped (Brunson & Weitzer, 2009; Levine & Small, 2008). Beyond the proximate damage caused, if these experiences happen in large numbers, vicarious knowledge of these incidents may then lead to undesirable effects on community perceptions of the police in the long run (Miller & D'Souza, 2015).

Conversely, the effect of pedestrian stops on feelings of police legitimacy may be dependent on the manner in which officers conduct stops and the purposes of the stop intervention. Stops that are conducted in a procedurally just manner, with attention paid to citizen participation, respect, neutrality, and trustworthy motives, may have the potential to increase positive police‐citizen contact and increase feelings of police legitimacy (Mazerolle et al., 2013; Tyler, 2004). Similarly, if the programmatic goal of a stop intervention aligns with the priorities of the community (i.e., gun seizures, drug crime reduction, etc.) it may further improve the perceived responsiveness of the police to community concerns.

### 1.4 Why it is important to do the review

Proactive policing tactics play an important role in crime prevention (Skogan & Frydl, 2004; Telep & Weisburd, 2012; Weisburd & Eck, 2004; Weisburd & Majmundar, 2018). However, the effects of proactive interventions vary greatly by the type of intervention and the manner in which the intervention is applied. Some tactics raise critical questions about the impacts of policing on the communities that they serve (Braga, Brunson, et al., 2019; Tyler et al., 2014).

Police have long felt that pedestrian stops can have an important general and specific deterrent value in preventing crime. Research evidence supporting this view began to develop in the 1990s with evaluations of police crackdowns (Sherman, 1990). There is evidence that many cities across the United States were using pedestrian stops as a key crime prevention tool (Gelman et al., 2007; White & Fradella, 2016), and indeed the use of pedestrian stops has often correlated with decreasing crime in major U.S. cities (Weisburd et al., 2014). But a rigorous assessment of crime prevention outcomes of pedestrian stops has not been developed to date. A key contribution of our review will be to identify whether pedestrian stops reduce crime, and if so to identify the magnitude of that impact.

As noted earlier, a number of scholars have documented negative impacts of pedestrian stops on citizens and communities. In recent years, pedestrian stop tactics have come under increased legal scrutiny. For example, a federal district court ruling in *Floyd v. City of New York* (2013) found the New York City Police Department's use of SQF unconstitutional on the basis of racial disparity. Similar lawsuits have been brought against other U.S. police departments during the past decade (American Civil Liberties Union, 2010), and the perceived abuse of stop and search powers has led to riots and legal challenges in several European countries as well (Lennon & Murray, 2018; Murray et al., 2020).

Due to these concerns, pedestrian stop tactics have become extremely controversial. Perhaps as a result of this controversy, recent years have seen the use of such stops decrease substantially in major cities such as New York and Philadelphia (McNeil, 2020; Weisburd et al., 2016), and in European countries such as England and Scotland (Lennon & Murray, 2018; Tiratelli et al., 2018). There has even been a growing call among many to do away with pedestrian stop tactics entirely (see Baker & Goldstein, 2012). Yet, existing reviews have often failed to find evidence of negative impacts on community evaluations of the police—though negative effects on people who are stopped has a stronger evidence base (e.g., see Weisburd & Majmundar, 2018). Thus, it is increasingly important to determine if pedestrian stops have a crime prevention impact, and whether they produce negative consequences for the people and communities affected by them. To date, no review has systematically assessed these outcomes or simultaneously considered them alongside each other. Such a review is critical for informed crime prevention policy that weighs all potential costs and benefits.

## 2 OBJECTIVES

Given that pedestrian stop tactics have garnered controversy and concern over their potential effects on crime (see Macdonald et al., 2016; Rosenfeld & Fornango, 2014; Weisburd et al., 2016), community (see Baker & Goldstein, 2012; Gelman et al., 2007; Miller & D'Souza, 2015; Tyler et al., 2014) and health‐related outcomes (Geller et al., 2014), the main objective of this review is to examine the impact of pedestrian stops across each of these areas. Specifically, this review will synthesise the effect of police‐initiated pedestrian stops on five discrete outcome groupings:

- Crime and disorder;
- Violence in police‐citizen encounters;
- Police misbehavior;
- Community outcomes including fear of crime and perceptions of police (legitimacy, trust, satisfaction, and effectiveness); and
- Health‐related outcomes.

Our second objective is to examine whether the effects of police‐initiated pedestrian stops vary according to potential moderating factors. Assuming data is sufficient, we will assess whether effect sizes vary significantly based on (a) research design (see Weisburd et al., 2001); (b) country in which the intervention took place; (c) size of the geographic area at which the intervention was applied (e.g., micro versus macro‐place); and (d) crime type of focus (e.g., violent versus drug crime). Where data is sufficient, and given the concerns related to the concentration of pedestrian stops among racial minorities (Fagan & Davies, 2000), we will also examine racial composition as a moderating factor. Ultimately, this review seeks to provide a comprehensive account of the effects of pedestrian stop tactics for policing agencies and city governments, allowing them to consider both the effects on crime and the community when deciding whether to pursue such tactics.

## 3 METHODS

### 3.1 Criteria for considering studies for this review

#### 3.1.1 Types of studies

For studies to be considered eligible for this review the evaluation must include a target area or group where pedestrian stops are heightened (or reduced) and a comparison area which reflects reduced use of pedestrian stops (or heightened use). We suspect that most studies will involve proactive police interventions in target areas where pedestrian stops are encouraged as a crime prevention tool, and where the control areas will receive standard policing interventions. At the same time, we will include studies which compare areas (such as beats or precincts), or cities, in which there are “natural” variations in the use of pedestrian stops. Such variation could be the result of variation in policies, or long term historical patterns in policing.

Randomized and quasi‐experimental research designs will be eligible for inclusion in the review (Campbell & Stanley, 1966; Cook & Campbell, 1979; Shadish et al., 2002). This inclusion threshold is adapted from the inclusion criterion in Global Policing Database protocol (Higginson et al., 2015, p. 47–48), which will be the primary search source for this review:.

- Randomized experimental designs (RCTs)
- The following “strong” quasi‐experimental designs:
  a. Regression discontinuity designs
  b. Matched control group designs with or without pre‐intervention baseline outcome measures (propensity or statistically matched)
  c. Short interrupted time‐series designs with control group (<25 pre‐ and 25 postintervention observations; Glass, 1997)
  d. Long interrupted time‐series designs with control group (≥25 pre‐ and postintervention observations; Glass, 1997)

- The following less rigorous quasi‐experimental designs:
  a. Unmatched control group designs with pre‐post intervention measures of the outcome which allow for difference‐in‐difference analyses
  b. Unmatched control group designs without preintervention measures of the outcome where the control group has face validity
  c. Raw unadjusted correlational designs where the variation in the level of the intervention is compared to the variation in the level of the outcome

We will include studies with “unmatched” control groups; for example, studies that compared a target area or group to the rest of the jurisdiction or group. As such, we will not restrict our review to quasi‐experiments with statistically matched control groups, though we will distinguish between statistical matching and “unmatched” groups in a moderator analysis.

#### 3.1.2 Types of participants

Given our interest in examining the impacts of pedestrian stops on crime, officer behavior, the community, and health outcomes, this review will include the following populations:

- Law enforcement officers (including any particular race, ethnicity, gender, etc.)
- Citizens (including citizens who are the subjects of pedestrian searches; and including any race, ethnicity, gender, etc.)
- Places (may be micro places such as street segments, clusters of addresses, police beats; meso‐places such as neighborhoods and communities; or macro‐places such as entire jurisdictions, police districts, etc.).

#### 3.1.3 Types of interventions

Studies that report on an evaluation of a proactive policing program in which the primary component of a policing intervention is pedestrian stops will be eligible for this review. This might include a general approach or policy throughout a policing jurisdiction or a coordinated program with the goal of reducing crime (though a comparison area would need to be identified to meet our inclusion criteria). In other words, pedestrian stops must be employed as a key element of a policing intervention to be considered for inclusion, but we will not exclude studies based on the a priori intent of that intervention. We will also include studies that compare different “regimes” of pedestrian stops absent a particular program or intervention. For example, jurisdictions or areas such as police precincts may be compared based on different levels of pedestrian stops that are the result of long term patterns of policing behavior.

The length, dosage, and goal of these stops may vary by study, but all will include increases in police‐initiated stops relative to standard policing or the absence of a police intervention. This review will not be limited to pedestrian stop interventions targeting specific types of crime or disorder (e.g., weapon and drug‐related crime), and will not be limited to any specific type of overarching policing tactic (e.g., hot spots policing, crackdowns, directed patrol, etc.). This review will exclude any studies employing pedestrian stops as a secondary component to other policing tactics (as the effects of the stop component would be difficult to isolate from the other components of the policing intervention).

#### 3.1.4 Types of outcome measures

If data are sufficient, our outcomes measures will be broken up into five primary groups with corresponding subgroups as listed below:

- Crime and disorder
- Incidents of violence in police‐citizen encounters
- Officer misbehavior
- Community outcomes
  a. Fear of crime
  b. Perceptions of police (legitimacy, satisfaction, trust, effectiveness)
- Health (physical health, mental health, mortality).

Crime and disorder‐related outcomes may be measured in a variety of ways (see Addington, 2010), including official crime measures (e.g., incident and arrest data, calls for service data, crime rates), unofficial crime measures (e.g., crimes reported by civilians), and systematic social observations of crime. We also note that crime and disorder categories may include (but are not limited to) property, drug, and violent offenses.

Incidents of violence in police‐citizen encounters will likely be measured through police use‐of‐force reports. We will attempt to be as discrete as possible, including capturing use‐of‐force that results from suspect resistance and varying levels of force when possible. We also note that this outcome is not necessarily a measure of unjustified use‐of‐force, and thus is distinguished from officer misbehavior. Officer misbehavior, rather, will be measured through formal citizen complaints or community surveys reporting on police abuse or violence. We also anticipate community surveys to be the primary method of data collection for studies measuring community outcomes of fear of crime and perceptions of police. While we do not anticipate many studies providing health related outcomes, those that do will likely utilize public surveys with people who have been subjected to pedestrian stops or community members living in areas experiencing a large quantity of pedestrian stops (see Geller et al., 2014; Sewell & Jefferson, 2016). It may also be possible for these studies to include official health outcomes, such as mental health and physical injury measured from hospital data, or other official data sources. For community and health related outcomes, subgroup information will be captured when possible (i.e., race, gender, socioeconomic status).

#### 3.1.5 Duration of follow‐up

We will not restrict eligibility to any particular follow‐up period. We expect to find studies with varying follow‐up periods, including those that measure the effects of pedestrian stops concurrently with the intervention and those that use a dedicated postintervention period. If there is notable variation in follow‐up periods across studies we will categorize and synthesize studies by length of follow‐up period, to include short follow‐up (<6 months), medium follow‐up (6 months to 1 year), and long follow‐up (>1 year).

#### 3.1.6 Types of settings

To be eligible for inclusion, the intervention must be targeted at a geographic area. Sometimes that will be on specific streets, others on large geographic units such as police beats and districts, or even the entire jurisdiction. Eligible studies may come from any geographic region and any racial, ethnic, or demographic makeup that otherwise meets our eligibility criteria. Eligible studies will also not be restricted to any written language and will be included regardless of publication status. We will use Google Translate to conduct title and abstract screening for any non‐English language studies, and we will also use Google Translate for the main text of any non‐English language articles that require full‐text review. Should this approach not produce unequivocal decision‐making, we will contact study authors to verify study eligibility and/or obtain missing data. We will include studies circulated between 1970 and 2018 in this review.

### 3.2 Search methods for identification of studies

#### 3.2.1 Electronic searches

The search will be led by the Global Policing Database (GPD) research team at the University of Queensland (Elizabeth Eggins and Lorraine Mazerolle) and Queensland University of Technology (Angela Higginson). The University of Queensland is home to the GPD (see http://www.gpd.uq.edu.au), which will serve as the main search location for this review. The GPD is a web‐based and searchable database designed to capture all published and unpublished experimental and quasi‐experimental evaluations of policing interventions conducted since 1950. There are no restrictions on the type of policing technique, type of outcome measure or language of the research (Higginson et al., 2015). The GPD is compiled using systematic search and screening techniques, which are reported in Higginson et al. (2015) and summarized in Appendices A and B. Broadly, the GPD search protocol includes an extensive range of search locations to ensure that both published and unpublished research is captured across criminology and allied disciplines.

To capture studies, we will use terms related to pedestrian stops to search the GPD corpus of full‐text documents that have been screened as reporting on a quantitative impact evaluation of a policing intervention. Specifically, we will use the following terms to search the title and abstract fields of the corpus of documents published from January 1970 and December 2019:

- stop*
- SQF
- frisk*
- search*
- “street pop*”
- “street check*”
- “street‐check*”

#### 3.2.2 Searching other sources

As the GPD search process discussed above isolates experimental and quasi‐experimental impact evaluations of policing interventions captured from academic and gray literature sources. We will also use additional strategies to supplement the GPD search. First, we will supplement the GPD search with additional sources from Japan, Korea, the Middle East, and Europe by consulting subject guides through the Duke University Library. Second, similar to recent reviews using the GPD (Hinkle et al., 2020; Lum et al., 2020; Mazerolle et al., 2020) we will perform hand searches in leading criminology journals for the 12 months prior to December 2019 search date to identify any recent studies not yet be indexed in the GPD and other databases.^1^ Third, we will conduct forward citation searches using Google Scholar and reference harvesting on all studies deemed eligible for inclusion as well as existing reviews on related topics (e.g., Braga, Turchan, et al., 2019; Koper & Mayo‐Wilson, 2012). Finally, after completing all searches, we will e‐mail our list of eligible studies to leading policing scholars knowledgeable in the area of pedestrian stop policing tactics. This list of scholars will be comprised of the lead authors of the studies deemed eligible for inclusion in this review as well as general policing experts identified by the research team. This may help to identify studies that the above searches missed, as these experts may be able to identify studies missing from our list, particularly studies in the gray literature such as dissertations and smaller research reports.

### 3.3 Data collection and analysis

#### 3.3.1 Description of methods used in primary research

The studies included in this review will use variations of a treatment versus comparison group design with postintervention measures. For example, Sherman and Rogan (1995) employed a nonequivalent control group quasi‐experimental design to evaluate an intervention targeting gun seizures in Kansas City (MO). The intervention was directed at a high‐crime patrol beat with a comparison beat selected based on similar pre‐existing crime rates and sociodemographic characteristics. The intervention consisted of increased patrol presence in the target beat, with particular emphasis on conducting safety frisks and traffic stops. Evaluation measures included the number of stops conducted, the number of guns seized, and various measures of violent crime.

It is likely, however, that the unit of analysis or original data collection in eligible studies will vary based on the outcome grouping. We anticipate studies evaluating crime and community outcomes to use places as the unit of analysis (ranging from street segments or addresses to neighborhoods, police districts, or even cities), while studies evaluating officer behavior or health‐related outcomes may use people as the unit of analysis. We anticipate that the majority of studies will be quasi‐experimental in nature, often selecting comparison areas or groups in posthoc fashion. A smaller number of studies may use randomization to assign areas to treatment or proactively match treatment and control groups/areas. Some studies may include multiple follow‐up periods.

#### 3.3.2 Criteria for determination of independent findings

Our unit of analysis will be the research study, such that each study will only be included once for each discrete outcome grouping. Where multiple reports concern the same underlying intervention, we will code all reports and choose the one deemed most complete from which to retrieve data (though information may be extracted from multiple reports when necessary or to provide more comprehensive coding). In cases where there are multiple jurisdictions included in a multisite study, we will code each of the sites as a separate study. Where a study or multiple reports concerning the same intervention contain multiple measurement time‐points, each will be coded and synthesized separately based on length of follow‐up period (e.g., short, medium, and long). Additionally, studies using clustered designs (i.e. assignment to conditions at higher‐level units than those being analyzed) often contain correlations between the units within a cluster. To account for the effects of this correlation on the standard error of the estimates, we will use the method described by Fu et al. (2013). To do so, however, the intra‐class correlation coefficient is needed, and where studies fail to report this, we will conduct sensitivity analyses at various ICC values (see Armstrong et al., 2018).

In cases where a study reports multiple outcomes from within the same outcome category (e.g., crime/disorder, officer behavior, community outcomes, etc.) and the same target group/area, we plan to calculate composite effect sizes with standard errors that are adjusted based on the dependence between the individual outcomes (see Borenstein et al., 2009). For instance, a study may measure the effects of pedestrian stops on both violent and property crime. In these cases, we will code all outcomes identified by the authors and provide the average effect sizes for that outcome category. The aggregation of effect sizes will be conducted using the method proposed by Borenstein et al. (2009), which corrects the variance estimate of the composite effect size based on the strength of the correlation between dependent outcomes. Where the correlations between outcomes cannot be determined we will contact study authors or estimate the correlation between outcomes at a range of plausible values. If very few studies report multiple outcomes from within the same outcome category, then we will conduct both a composite effect size analysis as well as an analysis of the most appropriate effect sizes based on an a priori decision‐rule. If possible, we will examine outcomes by crime type, and if a study measures the same specific outcome using two forms of data (e.g., calls for service and incident data measuring violent crime) we will average these effects to ensure that each study is only represented once.

For studies using multiple sites within the same jurisdiction, we will treat the results as multiple outcomes that will be averaged across sites. For example, if a study reports on the effects of pedestrian stops on crime in multiple target areas in one jurisdiction, we will treat this as one study with subunits.

#### 3.3.3 Selection of studies

The search results will first be screened on title and abstract for potential relevance to police stops. To begin, all screeners will review the same subset of 25 titles/abstracts to establish reliability. These 25 titles/abstracts will be examined by a third author, and where discrepancies exist, all study authors with discuss the discrepancies and provide guidance for further eligibility screening. The rest of the results will then be divided among screeners for abstract/title review and a random sample of 5% of each screener's exclusions will be checked by a third author for quality assurance. Cross‐checking will continue until rates of incorrect exclusions fall below 5% of all screenings, or the third author may consider rescreening all exclusions if a screener's decisions appear unreliable. If further questions regarding the eligibility of any particular titles/abstracts are raised, all study authors will discuss and determine the eligibility of the study in question at this initial stage.

All studies that are deemed potentially eligible after title/abstract review will then receive full‐text review. Before beginning full‐text review all screeners will once again review the same subset of 25 studies to establish reliability. The eligibility determinations for these 25 studies will be compared among screeners by a third author, and where discrepancies exist, all study authors will once again discuss and provide guidance for further screening. Any subsequent studies that generate uncertainty among screeners will receive full‐text review from the review authors to determine final eligibility, and a random sample of 5% of each screener's exclusions will be check by a third author.

#### 3.3.4 Data extraction and management

Once the final sample of eligible studies is determined, all studies will be double coded by Kevin Petersen (an author of this protocol) and another research assistant at George Mason University using the coding sheet in Appendix C. A third author will compile the coding for all studies and then check for consistency between both coders, and any discrepancies will be discussed among the entire review team. Where discrepancies in coding occur during the remainder of the coding process, all review authors will discuss these discrepancies and come to a final coding decision. All review authors will monitor study coding and have biweekly calls to discuss coding progress.

All eligible studies will be coded (see coding protocol attached in Appendix C) on a variety of criteria (including details related to them) including:

(a) Reference information (title, authors, publication etc.)
(b) Nature and description of site selection, group, targeted outcome, and so forth.
(c) Nature and description of selection of comparison group or period
(d) The unit of analysis
(e) The sample size
(f) Methodological type (randomized experiment or quasi‐experiment)
(g) A description of the pedestrian stop intervention
(h) Dosage intensity and type
(i) Implementation difficulties
(j) The statistical test(s) used
(k) Reports of statistical significance (if any)
(l) Effect size/power (if any)
(m) The conclusions drawn by the authors.

#### 3.3.5 Assessment of risk of bias in included studies

Studies deemed eligible for inclusion in the meta‐analysis will be assessed for risk of bias using the Cochrane randomized and non‐randomized risk of bias tools, based on study design (Higgins et al., 2019). Coding items consistent with these tools will allow for the categorization of randomized experiments into low risk, some concerns, or high‐risk classifications, and quasi‐experimental designs into low risk, moderate risk, serious risk, or critical risk classifications. We anticipate that the risk of bias tools may need to be adapted to better capture the issues arising in our studies, particularly those that utilize geographic areas as units of analysis. We plan to modify these tools during the course of the review; however, these modifications will be dictated by the issues that are identified as salient upon review of our eligible studies. Risk of bias ratings across eligible studies will be presented in tabular and narrative format and sensitivity analyses may be performed depending on the level of bias noted in our eligible studies.

#### 3.3.6 Measures of treatment effect

For eligible place‐based studies containing the requisite data, effect sizes will be calculated using a log relative incident rate ratio (RIRR). We anticipate that many of our studies will include place‐based count data. While prior meta‐analyses using this form of data have often calculated effect sizes using an odds ratio (OR) that is then converted to a standardized mean difference effect size (Cohen's *d*; see Braga, Turchan, et al., 2019), Wilson (2021) suggests that Cohen's *d* effect sizes are not necessarily comparable across studies when computed using place‐based count data, and that Cohen's *d* values calculated using the above conversion are not comparable to those calculated directly through conventional means (see Braga & Weisburd, 2020; Hinkle et al., 2020; for evidence of these problems in meta analyses). The log RIRR approach, when exponentiated, produces an easily interpretable percent change for the treatment group relative to the control group and has been used in a recent Campbell systematic review of problem‐oriented policing, which analyzed studies with similar forms of data to those we anticipate in this review (see Hinkle et al., 2020). The general equation for the log RIRR is represented below (see Wilson, 2021):

$$\log\text{RIRR}=\log\left(\frac{X_{11}X_{00}}{X_{01}X_{10}}\right).$$

With the first subscript representing treatment (1) or control (0) grouping, and the second subscript representing pre (0) or postintervention (1) time period. The product of this equation provides the change in the counts (or rates) of incidents from pre to post intervention for the treatment group relative to the control group, and thus can be compared across studies with varying geographic units and evaluation periods. The standard error of the log RIRR can then be initially calculated using the following equation:

$${\mathrm{se}}_{\log(\mathrm{RIRR})}=\sqrt{\frac{1}{X_{11}}+\frac{1}{X_{10}}+\frac{1}{X_{01}}+\frac{1}{X_{00}}}.$$

Given that the RIRR assumes a Poisson distribution, however, Wilson (2021) suggests the following correction for overdispersion:

$$Ф=\left(\frac{1}{\Sigma_{n_{k}}-4}\right)\Sigma \frac{s_{k}^{2}(n_{k}-1)}{{\bar{X}}_{k}},$$

where ${\overset{®}{X}}_{k}$ is the average count for treatment and control area across both pre and postintervention time periods, $S_{k}$ is the standard deviation for each average count, and $n_{k}$ is the number of counts (contributing to the mean) for both treatment and control groups across pre and postintervention periods (Wilson, 2021). Experience in prior reviews (e.g., see Braga & Weisburd, 2020; Hinkle et al., 2020) suggest that the data for estimating over‐dispersion is likely not available in many studies. Because of this we will use the method proposed by Farrington et al. (2007) to estimate confidence intervals for the produced statistics. However, we will conduct a sensitivity analysis of our overall findings by calculating the Farrington et al. and Wilson estimates for the overall mean effects for studies that data allow and compare summary findings. If a sufficient number of studies are available we will also apply the mean correction using the Wilson approach to all studies and compare the overall findings using both methods.

For studies providing binary outcomes or prevalence data (e.g., the presence/absence of an injury or condition), effect sizes will be calculated using a risk ratio, which largely follows the equation for the RIRR provided above and should allow for easier comparison of these effects with those of place‐based count outcomes (Wilson, 2021). Effect sizes for individual‐level outcomes, such as citizen surveys, measured on a Likert‐scales will be calculated using Cohen's *d*.

#### 3.3.7 Dealing with missing data

Where otherwise eligible studies lack the requisite data to calculate effect sizes, make determinations on risk of bias, or other salient coding areas, we will contact the study authors in attempt to obtain the necessary data. If this is unsuccessful, studies will be excluded from the meta‐analysis if effect sizes cannot be calculated. However, these studies will still be noted and included in our narrative descriptions to avoid the introduction of bias from lack of reporting.

#### 3.3.8 Assessment of heterogeneity

We will examine the presence of heterogeneity in effect sizes across studies using the *Q* statistic. Additionally, we will examine the extent of such heterogeneity using $I^{2}$ and τ^2^ statistics. Heterogeneity will also be explored using the variables listed in the subgroup analysis section.

#### 3.3.9 Assessment of reporting biases

We will use traditional methods to test for the sensitivity of the findings to publication/reporting bias in the experimental and quasi‐experimental studies. These methods will include a comparison of the mean effect size for published and unpublished studies, a trim‐and‐fill analysis, and funnel plot.

#### 3.3.10 Data synthesis

Data permitting, effect sizes for conceptually analogous studies will be combined using inverse‐variance weighted meta‐analysis. Given that our studies will likely be conducted in various settings, with various forms of pedestrian stop interventions, and with various types of samples and corresponding populations, we will assume a random effects model. In other words, we acknowledge that the true effect of pedestrian stops may differ based on the unit of analysis/intervention and the specifics of the strategy, and thus believe that a mixed effects model is most suitable for analyzing the effectiveness of pedestrian stop interventions. Data synthesis will be developed using Comprehensive Meta Analysis software, https://www.meta-analysis.com, and the method‐of‐moments random effects estimator will be used.

#### 3.3.11 Subgroup analysis and investigation of heterogeneity

As noted above, we hope to examine factors moderating the effect of pedestrian stops on our outcome measures. Though it is difficult to know what moderator analyses will be possible given the data, at minimum we hope to assess the differential effects of pedestrian stops on research design, location where the intervention took place, size of the geographic area at which the intervention was applied (e.g., micro versus macro‐place), crime type of focus (e.g., violent versus drug crime), and racial or ethnic composition of the communities in the study or the persons stopped. Additionally, to the extent possible, we will conduct a moderator analysis based on the intent of the intervention. In other words, stop interventions that were a key component of an intervention with a priori intent to deter or prevent crime will be analysed separately from other studies, such as those in which intent cannot be determined or those with posthoc analysis of differing stop policies or numbers of stops conducted. If our search identifies enough data to assess these effects we will use the analog to the ANOVA method of moderator analysis (see Lipsey & Wilson, 2001) for categorical moderator variables and meta‐analytic regression analysis for continuous moderator variables or analyses involving multiple moderators. Depending on our results, we may wish to conduct additional posthoc moderator analyses. If so, we will clearly delineate a priori analyses from those that were determined posthoc.

#### 3.3.12 Sensitivity analysis

In addition to including sensitivity analyses using smallest, largest, and combined effect sizes, publication biases, and various ICC levels (if necessary), we will also conduct sensitivity analyses excluding studies with significant risk of bias.

#### 3.3.13 Treatment of qualitative research

We do not plan to include qualitative research in this review.

## ROLES AND RESPONSIBILITIES

- Content: David Weisburd, Kevin Petersen, Taryn Zastrow, Robert Davis, Lorraine Mazerolle, and Elizabeth Eggins.
- Systematic review methods: David Weisburd, Kevin Petersen, Taryn Zastrow, Lorraine Mazerolle, and Elizabeth Eggins.
- Statistical analysis: David Weisburd, Kevin Petersen, Lorraine Mazerolle, and Elizabeth Eggins.
- Information retrieval: Kevin Petersen, Taryn Zastrow, Lorraine Mazerolle, Elizabeth Eggins, and research assistants.

## DECLARATIONS OF INTEREST

David Weisburd has conducted a number of evaluations of hot spots policing and has written an article on SQFs showing some degree of effectiveness. He was also the Chair of the National Academy of Sciences panel on proactive policing, which suggested effectiveness of targeted pedestrian stops (though little impact for broadly focused policies). David Weisburd would be comfortable with outcomes that run counter to these prior findings. He is a member of the Campbell Collaboration Crime and Justice Coordinating Group. To manage potential conflicts of interest, David Weisburd will not be involved in the editorial or formal approval process for this protocol or the subsequent review. Kevin Petersen and Taryn Zastrow have not conducted evaluation research or published on the effectiveness of pedestrian stops. Neither author would be uncomfortable with any results produced by the review. Robert Davis has not published prior work in this area. Lorraine Mazerolle has conducted a number of evaluations of policing interventions, some of which have contained police stops as one component of the intervention. She is also the Co‐Chair of the Campbell Collaboration Crime and Justice Coordinating Group. To manage these potential conflicts of interest, Lorraine Mazerolle will not be involved in the editorial or formal approval process for this protocol or the subsequent review, nor will she independently decide on study eligibility, code studies, or conduct statistical or risk of bias analyses. Elizabeth Eggins has not been involved in any evaluations of police‐initiated pedestrian police‐stops. Elizabeth Eggins is the Managing and Associate Editor for the Campbell Crime and Justice Coordinating Group and so will not be involved in the editorial or formal approval process for this protocol or the subsequent review.

## PRELIMINARY TIMEFRAME

The review process will adhere to the following schedule:

**Table:** 

Submission of protocol	Fall 2020
Completion of GPD search	Spring 2021
Coding of eligible studies	Spring 2021 to Summer 2021
Submission of completed report	Winter 2021

## PLANS FOR UPDATING THE REVIEW

The authors expect to update the review every 5–10 years depending on a sense of trends for experimental and quasi‐experimental evaluations and available funding and support.

## Figures and Tables

**Figure A1:**
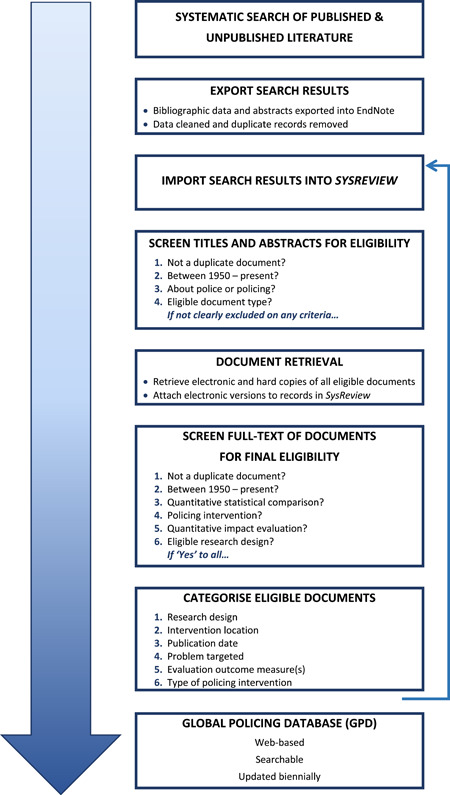
GPD systematic compilation process

## Footnotes

1 These journals will include: *Cambridge Journal of Evidence‐Based Policing, Criminology, Criminology and Public Policy, Justice Quarterly, Journal of Research in Crime and Delinquency, Journal of Criminal Justice, Police Quarterly, Policing, Police Practice and Research, British Journal of Criminology, Journal of Quantitative Criminology, Crime and Delinquency, Journal of Criminal Law and Criminology, Policing and Society*.

## APPENDIX A GPD SYSTEMATIC SEARCH STRATEGY 2 Appendices A and B are taken directly from Higginson, A., Eggins, E., Mazerolle, L. and Stanko, E. (2015). *The Global Policing Database [Database and Protocol]*.

### Search Terms

To ensure optimum sensitivity and specificity, the GPD search strategy utilizes a combination of free‐text and controlled vocabulary search terms. Because controlled vocabularies and search capabilities vary across databases, the exact combination of search terms and field codes are adapted to each database. Final search syntax for each location will be reported in the final review.

The free‐text search terms for the GPD are provided in Table A1 and are grouped by substantive (i.e., some form of policing) and evaluation terminology. Although the search strategy may vary slightly across search locations, it follows a number of general rules:

**Table A1:** Free‐text search terms for the GPD systematic search

Policing search terms	Evaluation search terms			
police policing “law*enforcement” constab* detective* sheriff*	analy* ANCOVA ANOVA “ABAB design” “AB design” baseline causa* “chi#square” coefficient* “comparison condition*” “comparison group*” “control condition*” “control group*” correlat* covariat* “cross#section*”	data effect* efficacy eval* experiment* hypothes* impact* intervent* interview* longitudinal MANCOVA MANOVA “matched group” measure* “meta‐analy*” “odds#ratio*	outcome* paramet* “post‐test” posttest “post test” predict* “pre‐test” pretest program* “propensity score*” quantitative “quasi#experiment*” questionnaire* random* RCT regress*	result* “risk#ratio*” sampl* “standard deviation*” statistic* studies study survey* “systematic review*” “t#test*” “time#series” treatment* variable* variance

- Search terms are combined into search strings using Boolean operators “AND” and “OR.” Specifically, terms within each category are combined with “OR,” and categories will be combined with “AND.” For example: (police OR policing OR “law#enforcement”) AND (analy* OR ANCOVA OR ANOVA OR …).
- Compound terms (e.g., law enforcement) are considered single terms in search strings by using quotation marks (i.e., “law*enforcement”) to ensure that the database searches for the entire term rather than separate words.
- Wild cards and truncation codes are used for search terms with multiple iterations from a stem word (e.g., evaluation, evaluate) or spelling variations (e.g., evaluat* or randomi#e).
- If a database has a controlled vocabulary term that is equivalent to “POLICE,” the term is combined in a search string that includes both the policing and evaluation free‐text search terms. This approach ensures that the search retrieves documents that do not use policing terms in the title/abstract but have been indexed as being related to policing in the database. An example of this approach is the following search string: (((SU: “POLICE”) OR (TI,AB,KW: police OR policing OR “law*enforcement”)) AND (TI,AB,KW: intervention* OR evaluat* OR compar* OR …)).
- For search locations with limited search functionality, a broad search that uses only the policing free‐text terms is implemented.
- Multidisciplinary database searches are limited to relevant disciplines (e.g., include social sciences but exclude physical sciences).
- Search results are refined to exclude specific types of documents that are not suitable for systematic reviews (e.g., newspapers, front/back matter, book reviews).

### Search Locations

To reduce publication and discipline bias, the GPD search strategy adopts an international scope and involves searching for literature across a number of disciplines (e.g., criminology, law, political science, public health, sociology, social science and social work). The search captures a comprehensive range of published (i.e., journal articles, book chapters, books) and unpublished literature (e.g., working papers, governmental reports, technical reports, conference proceedings, dissertations) by implementing a search strategy across bibliographic/academic, gray literature, and dissertation databases or repositories.

**Table A2:** GPD search locations and protocol (January 1st, 1950, to December 2018)

Indexed and academic databases		Content coverage fed into OSID?	Full or modified search?	Search modifications
ProQuest	Criminal Justice	Yes	Full	None
	Dissertation and Theses Database Global	Not Available	Modified	Social Sciences subset
	Political Science	Yes	Full	None
	Periodical Archive Online	Yes	Full	None
	Research Library	Yes	Modified	Social Sciences subset
	Social Science Journals	Yes	Full	None
	Sociology	Yes	Modified	Search 2 unique journal titles and non‐serial content only
	Applied Social Sciences Index and Abstracts	Yes	Full	None
	International Bibliography of the Social Sciences	Yes	Full	None
	Public Affairs Information Service	Yes	Full	None
	Social Services Abstracts	Yes	Modified	Search 5 unique journal titles and non‐serial content only
	Sociological Abstracts	Yes	Full	None
	Worldwide Political Sciences Abstracts	Yes	Modified	Search 9 unique journal titles and non‐serial content only
EBSCO	Academic Search Premier	Yes	Full	None
	Criminal Justice Abstracts	Yes	Full	None
	EconLit	Yes	Full	None
	MEDLINE with Full‐Text	Yes	Full	None
	Social Sciences Full‐Text	Yes	Full	None
OVID	International Political Science Abstracts	Not Available	Full	None
	PsycARTICLES	Yes	Modified	Search 4 unique journal titles only
	PsycEXTRA	Not Available	Full	None
	PsycINFO	Yes	Full	None
	Social Work Abstracts	Not Available	Full	None.
Web of Science	Current Contents Connect – Social and Behavioural Sciences Edition	Yes	Modified	Search 1 unique journal title and non‐serial content only
	Book Citation Index (Social Sciences and Humanities)	Not Available	Full	None
	Conference Proceedings Citation Index (Social Sciences and Humanities)	Not Available	Full	None
	Social Science Citation Index	Yes	Full	None
Informit	Australian Attorney General Information Service	Yes	Full	None
	Australian Criminology Database (CINCH)	Yes	Full	None
	Australian Federal Police Database	Yes	Full	None
	Australian Public Affairs Full‐Text	Yes	Full	None
	DRUG	Yes	Full	None
	Health & Society Database	Yes	Modified	Search unique journal titles and non‐serial content only
	Humanities and Social Sciences Collection	Yes	Full	None
Gale‐Cengage	Expanded Academic ASAP	Yes	Full	None
STANDALONE & OPEN ACCESS DATABASES	Cambridge Journals Online	Yes	Modified	Search 4 unique journal titles in Law and Political Science collections and full search of Social Studies collection
	Directory of Open Access Journals	Yes	Full	None
	HeinOnline	Yes	Modified	Law Journals Online collection only
	JSTOR	Yes	Modified	Search unique titles across the Law, Political Science, Public Health, Public Policy, Social Work and Sociology collections only. The Criminal Justice collection had no unique content and so will be excluded from the search. Only 10% of content in this database have abstracts and a full‐text search returns >250,000 results because of inability to construct complex search strings. Therefore, a modified search of the unique titles across these collections will be more pragmatic than a full search of the database
	Oxford Scholarship Online	Yes	Full	None
	Sage Journals Online and Archive (Sage Premier)	Yes	Modified	Search 5 unique journal titles and non‐serial content only
	ScienceDirect	Yes	Full	None
	SCOPUS	Yes	Full	None
	SpringerLink	Yes	Full	Although this database has low uniqueness when combined with the full set of databases, a full search using only the policing search terms will be more pragmatic than a modified search on unique titles because of the restricted search functionality of this database
	Taylor & Francis Online	Yes	Modified	Although this database has low uniqueness when combined with the full set of databases, a full search using only the policing search terms will be more pragmatic than a modified search on unique titles because of the restricted search functionality of this database
	Wiley Online Library	Yes	Full	None
	California Commission on Peace Officer Standards & Training Library	No	Full	None
	Cochrane Library	No	Full	None
	CrimeSolutions.gov	No	Full	None
	Database of Abstracts of Reviews of Effectiveness (DARE)	No	Full	None
	FBI – The Fault (Reports and Publications)	No	Full	None
	Evidence‐Based Policing Matrix	No	Full	None
	International Initiative for Impact Evaluation Database (3ie)	No	Full	None
	National Criminal Justice Reference Service	No	Full	None
	Safety Lit Database	No	Full	None
	Australian Institute of Criminology	No	Full	None
	Bureau of Police Research and Development (India)	No	Full	None
	Canadian Police Research Catalogue	No	Full	None
	Centre for Problem‐Oriented Policing	No	Full	None
	College of Policing (including POLKA and Crime Reduction Toolkit)	No	Full	None
	European Police College (CEPOL)	No	Full	None
	Evidence for Policy and Practice Information and Coordinating Centre	No	Full	None
	National Research Institute of Police Science (Japanese)	No	Full	None
	Office of Community Oriented Policing Services	No	Full	None
	Police Executive Research Forum (US)	No	Full	None
	Police Foundation (US)	No	Full	None
	Tasmania Institute of Law Enforcement Studies (Australia)	No	Full	None
	Policing Online Information System (POLIS, Europe)	No	Full	None
	Scottish Institute for Policing Research	No	Full	None
	Centre of Excellence in Policing and Security (Australian, now archived)	No	Full	None

It is noted that there is substantial overlap of the content coverage between many of the databases. Therefore, the *Optimal Searching of Indexing Databases* (OSID) computer program (Neville & Higginson, 2014) has been used to analyse the content crossover for all databases that have accessible content coverage lists. OSID analyses the content coverage and creates a search location solution that provides the most comprehensive coverage via the least number of databases. Another advantage of using OSID when designing a search strategy is the reduction in the number of duplicates that would need to be removed prior to the screening phase. Databases with >10 unique titles are searched in full, whereas databases with ≤10 unique titles were searched only the unique titles and any non‐serial content (e.g., reports, conference proceedings). Where a modified search of a database would be more labor‐intensive than a full search and export results, a full search of the database is conducted. The final search locations and solutions are reported in Table A2.

## APPENDIX B GPD SYSTEMATIC COMPILATION STRATEGY

### Inclusion Criteria

Each record captured by the GPD systematic search must satisfy all inclusion criteria to be included in the GPD: timeframe, intervention and research design. There are no restrictions applied to the types of outcomes, participants, settings or languages considered eligible for inclusion in the GPD.

#### Types of interventions

Each document must contain an impact evaluation of a policing intervention. Policing interventions are defined as some kind of a strategy, program, technique, approach, activity, campaign, training, directive, or funding/organizational change that involves police in some way (other agencies or organizations can be involved). Police involvement is broadly defined as:

- Police initiation, development or leadership
- Police are recipients of the intervention or the intervention is related, focused or targeted to police practices
- Delivery or implementation of the intervention by police.

#### Types of study designs

The GPD includes quantitative impact evaluations of policing interventions that utilize randomized experimental (e.g., RCTs) or quasi‐experimental evaluation designs with a valid comparison group that does not receive the intervention. The GPD includes designs where the comparison group receives “business‐as‐usual” policing, no intervention or an alternative intervention (treatment‐treatment designs).

The specific list of research designs included in the GPD are as follows:

- Systematic reviews with or without meta‐analyses
- Cross‐over designs
- Cost‐benefit analyses
- Regression discontinuity designs
- Designs using multivariate controls (e.g., multiple regression)
- Matched control group designs with or without preintervention baseline measures (propensity or statistically matched)
- Unmatched control group designs with pre‐post intervention measures which allow for difference‐in‐difference analysis
- Unmatched control group designs without pre‐intervention measures where the control group has face validity
- Short interrupted time‐series designs with control group (<25 pre‐ and 25 postintervention observations)
- Long interrupted time‐series designs with or without a control group (≥25 pre‐ and postintervention observations)
- Raw unadjusted correlational designs where the variation in the level of the intervention is compared to the variation in the level of the outcome

The GPD excludes single group designs with pre‐ and postintervention measures as these designs are highly subject to bias and threats to internal validity.

### Systematic Screening

To establish eligibility, records captured by the GPD search progress through a series of systematic stages which are summarized in Figure A1, with additional detail provided in the following subsections.

All research staff working on the GPD undergo standardized training before beginning work within any of the stages detailed below. Staff then complete short training simulations to enable an assessment of their understanding of the GPD protocols and highlight any areas for additional training. In addition, random samples of each staff's work are regularly cross‐checked to ensure adherence to protocols. Disagreements about screening decisions between staff are mediated by either the project manager or GPD chief investigators.

#### Title and abstract screening

After removing duplicates, the title and abstract of records captured by the GPD systematic search is screened by trained research staff to identify potentially eligible research that satisfies the following criteria:

- Document is dated between 1950 and present
- Document is unique (i.e., not a duplicate)
- Document is about police or policing
- Document is an eligible document type (e.g., not a book review)

Records are excluded if the answer to any one of the criteria is unambiguously “No,” and will be classified as potentially eligible otherwise. Records classified as eligible at the title and abstract screening stage progress to full‐text document retrieval and screening stages.

#### Full‐text eligibility screening

Wherever possible, a full‐text electronic version of an eligible record is imported into *SysReview* (review management software; Neville & Higginson, 2014). For records without an electronic version, a hardcopy of the record is located to enable full‐text eligibility screening. The full‐text of each document is screened to identify studies that satisfy the following criteria:

- Document is dated between 1950 and present
- Document is unique
- Document reports a quantitative statistical comparison
- Document reports on policing evaluation
- Document reports in a quantitative impact evaluation of a policing intervention
- Evaluation uses an eligible research design

## APPENDIX C PEDESTRIAN STOPS META ANALYSIS CODING SHEET

### Reference Information

1. Document ID: __ __ __ __
2. Study author(s): ____________________
3. Are multiple publications/reports associated with this intervention?

1. Yes
2. No

3. If yes, list secondary/additional study author(s):___________
4. Study title(s) associated with intervention: _______________________
5. Primary publication type(s): ______

1. Book
2. Book chapter
3. Journal article (peer reviewed)
4. Thesis or doctoral dissertation
5. Government report (state/local)
6. Government report (federal)
7. Police department report
8. Technical report
9. Conference paper
10. Award submission
11. Other (specify)

4. Specify (Other)_____________________
5. Publication date (year): ______________
6. Journal Name: ____________________
7. Journal Volume: _______________
8. Journal Issue: ____________
9. Date range of research (i.e., time span covered by research from pre to post‐intervention follow‐up):

Start: ____________

Finish: ____________

8. Source(s) of funding for study: ___________________
9. Country of publication: ___________________
10. Date coded: ___________
11. Coder's Initials: __ __ __

### Describing the Intervention(s)

12. Were pedestrian stops the only strategy applied during the intervention?

1. Yes
2. No
3. Unclear

12. Which of the following best describes the type of intervention? (Select all that apply)

1. Police crackdown using pedestrian stops as a major component
2. Hot spot policing intervention using pedestrian stops as a major component
3. Directed patrol intervention using pedestrian stops as a major component
4. Random preventative patrol intervention using pedestrian stops as a major component
5. Disorder policing intervention using pedestrian stops as a major component
6. Problem‐oriented policing intervention using pedestrian stops as a major component
7. Other (specify)

12. Specify (Other) _____________
13. What was the target of the pedestrian stops? (Select all that apply)

1. Weapons
2. Violent crime generally
3. Drugs
4. Gangs
5. General crime at specific places
6. N/A (no specific target)
7. Other (specify)

13. Specify (Other) _______________

14. What other enforcement actions took place during the intervention? (Select all that apply)______
15. Search warrants/raids
16. Disorder enforcement
17. POP approaches
18. Misdemeanor arrests
19. Felony arrests
20. Traffic stops/vehicle checks
21. No enforcement activities noted
22. Other
23. Specify (Other) ___________
24. Describe the nature of the intervention, including the rationale for using stops and the specific activities that officers were told to engage in (if provided). ________________________________________________________________

____________________________________________________________________________________________________________________________________________________________

16. Which of the following best describe the dosage of the police intervention?

(check all that apply)

1. Officers from patrol assigned to conduct stops full‐time
2. Officers from patrol assigned to conduct stops part‐time
3. Officers from special unit assigned to conduct stops full‐time
4. Officers from special unit assigned to conduct stops part‐time
5. Officers from patrol conduct stops during downtime (e.g., between calls)
6. Officers from special unit conduct stops during downtime
7. Other (specify)

16. Specify (Other) ___________________
17. Briefly describe the intensity/dosage of the police intervention (i.e. the amount of street stops and/or searches that were conducted).

________________________________________________________________

________________________________________________________________

________________________________________________________________

16. Please provide a “baseline”, for example per officer, per shift etc.

________________________________________________________________

________________________________________________________________

17. At what level of the police department was the response implemented? _____

1. Entire department/all officers involved
2. Certain precincts/districts involved
3. Special unit (e.g., hot spots unit) involved
4. Select few officers in specific area involved
5. Other (specify)
6. N/A (not mentioned)

17. Specify (Other)___________________
18. At what type of crime or disorder is the intervention directed? (select all that apply)

1. All offense types
2. Property offenses
3. Violent offenses
4. Drug offenses
5. Weapons offenses
6. Sexual offenses
7. Disorder offenses
8. Other (specify)
  18. Specify (Other ___________________________________
  19. At what geographic area was the pedestrian stops intervention targeted? _______
  20. Micro place (e.g., hot spot)
  21. Meso area (e.g., neighborhoods/police beats)
  22. Large area (e.g., entire city)
  23. Other (specify)
  24. Specify (Other) ___________________________
  25. Was the demographic (i.e. racial/ethnic and gender) composition of the targeted area discussed?
  26. Yes
  27. No
  28. If yes, briefly describe the information provided and the page number is it provided on. _________________________
  29. Was the socioeconomic status of the targeted area discussed?
  1. Yes
  2. No
  21. If yes, briefly describe the information provided and the page number it is provided on.
  __________________________
  22. What was the nature of the comparison or control condition?
  23. Standard policing
  24. No treatment
  25. Alternative policing intervention
  26. Other (specify)
  27. Specify (Other) ______________________
  28. If an alternative policing intervention was employed in the control/comparison area, which of the following best describes the type of intervention? (Select all that apply)
  29. Hot spot policing intervention
  30. Directed patrol intervention
  31. Random preventative patrol intervention
  32. Problem‐oriented policing intervention
  33. Community policing intervention
  34. Other (specify)
  35. Specify (Other) ___________________________

23. Briefly describe the nature of the comparison or control condition

______________________________________________________

______________________________________________________

24. Was the demographic (i.e. racial/ethnic and gender) composition of the control/comparison area discussed?

1. Yes
2. No

24. If yes, briefly describe the information provided and the page number is it provided on. _________________________
25. Was the socioeconomic status of the control/comparison area discussed?

1. Yes
2. No

25. If yes, briefly describe the information provided and the page number it is provided on.

__________________________

### Implementation of Intervention

26. What did the evaluation indicate about the implementation of the response? _____

1. There were no reported implementation issues
2. There were minor implementation issues
3. There were more substantial implementation issues
4. There were major implementation issues/the project was not implemented as planned
5. Unclear/no process evaluation included

27. If the process evaluation indicated there were problems with implementation of the response, describe these problems:

__________________________________________________________________________________________________________________________________________________________________________________________________________________________________________

### Location of the intervention

28. Country where study was conducted: __________________
29. City (and state/province, if applicable) where study was conducted: _________________

*The following questions refer to the area receiving treatment*:

30. Geographic area receiving treatment (may differ from area originally targeted): _______________________
31. Micro place (street segments/blocks)
32. Neighborhood/police beat
33. Police district/precinct
34. Entire city
35. Other (specify)
36. Specify (Other)___________________
37. What is the exact geographic area receiving treatment? ______________________________________________________________________________
38. Describe any information provided about the nature of the site selection.

_______________________________________________________________________________

*The following refer to the area not receiving treatment*

33. Geographic area NOT receiving treatment: ______

1. Micro place (street segments/blocks)
2. Neighborhood/police beat
3. Police district/precinct
4. Entire city
5. Other (specify)

33. Specify (Other)___________________
34. What is the exact geographic area not receiving treatment? ________________________________________________________________________________________________________________________________
35. Described any information provided about the nature of the control/comparison group selection

________________________________________________________________________________________________________________________________

### Methodology/Research design

36. Length of pre‐intervention study period______
37. Length of intervention study period_________
38. Length of post‐intervention study period______
39. Is there a secondary follow‐up period for this intervention? (*Note that for each additional follow‐up period, a separate coding sheet is required)*

1. Yes
2. No

38. Type of study: _____

1. Randomized experiment
2. Block randomized experiment
3. Nonequivalent control group (quasi‐experimental)
4. Other (specify)

38. Specify (Other)___________________
39. What process was used to assign study units to treatment or comparison conditions?

1. Simple random assignment
2. Random assignment from within blocks or pairs
3. Identification of matching areas or persons through regression analyses (e.g., propensity score matching)
4. Statistical tests of mean differences among demographic and other relevant variables
5. Comparison of descriptive statistics with no statistical test of differences across groups
6. Comparison to the rest of a jurisdiction or population that did not receive the treatment
7. Other (specify)

39. Specify (Other)___________________
40. Was multiple outcome measurement used?

1. Yes analyzed multiple time periods in a single analysis (e.g., statistical modeling with multiple dummy variables for time).
2. Yes conducted a time series statistical analysis
3. No (e.g., single pre/post observations)
4. Unclear
5. Other (Specify)

40. Specify (other) ____________________________
41. Explain any other measures that were taken to control for the influence of potentially confounding variables or to strengthen the internal validity of the study's results.____________________________________________________________________________________________________________________________________________________
42. Were any sources of non‐equivalence or bias reported or implied in the application of the intervention or its analysis (i.e. threats to internal validity)?

Yes

No

41. If yes, what sources of nonequivalence or bias were identified? (check all that apply and explain)
42. Extraneous events or factors occurring during the intervention period; historical artifacts
43. Selection of treatment area based on high baseline crime rate
44. Measurement confounds (measure changes over time)
45. Differential attrition, breakdown of randomization, or contamination of control group
46. Pre‐test analyses indicated nonequivalence between treatment and control groups
47. Statistical analyses failed to adjust for nonequivalence at baseline
48. Inappropriate statistical analysis for design
49. Any outcomes measured by reporters that did not have corresponding outcome measures in the results
50. Other threats to internal validity (specify)
51. Explain any yes responses checked in 31b.

______________________________________________________________________________________________________________________________________________________________________________________________________

41. If yes, did the researchers discuss the implications of the bias for their findings?
42. Yes
43. No
44. Did the researcher assess the quality of the data collected?
45. Yes
46. No
47. Did the researcher(s) express any concerns over the quality of the data?

1. Yes
2. No
  43. If yes, explain ____________________________________________________________________________________________________________________________________________________________
  ***Outcomes reported** (Note that for each outcome, a separate coding sheet is required. Outcomes may include crime/disorder, police‐citizen violence, police misbehaviour, community outcomes, or health‐related outcomes)*

44. How many outcomes are reported in the study? _____
45. What is the specific outcome recorded on this coding sheet?

_______________________________________________________________

46. Was it the primary outcome of the study? _______

1. Yes
2. No
3. Can't tell/researcher did not prioritize outcomes

47. Was this initially intended as an outcome of the study? ______

1. Yes
2. No (explain)
3. Can't tell

47. If no, explain why:

________________________________________________________________________________________________________________________________

### Dependent Variable

48. What type of data was used to measure the outcome covered on this coding sheet? ________________________________

1. Official data
2. Researcher observations
3. Self‐report surveys
4. Other (specify)

48. Specify (Other)___________________
49. If official data was used, what specific type(s) of data were used? (Select all that apply)

1. Calls for service (911 calls)
2. Arrests
3. Incident reports
4. Level of citizen complaints
5. Use of force reports
6. Official medical information
7. Other (specify)
8. N/A (official data not used)

49. Specify (Other)___________________
50. If researcher observations were used, what types of observations were taken? (Select all that apply)

1. Physical observations (e.g., observed urban blight, such as trash, graffiti)
2. Social observations (e.g., observed disorder, such as loitering, public drinking)
3. Other observations (specify)
4. N/A (researcher observations not used)

50. Specify (Other)___________________
51. If self‐report surveys were used, who was surveyed? (Select all that apply)

1. Residents/community members
2. Business owners
3. Elected officials
4. Government/social service agencies
5. Other (specify)
6. N/A (self‐report surveys not used)

51. Specify (Other)___________________

**Effect size/Reports of statistical significance**

***Sample size***

52. Based on the unit of analysis for this outcome, what is the total sample size in the analysis? ________
53. What is the total sample size of the treatment group (group that receives the response)? _______
54. What is the total sample size of the control group (if applicable)? _____
55. Was attrition a problem in the analysis for this outcome?

1. Yes
2. No

55. If attrition was a problem, provide details (e. g. how many cases lost and why they were lost).

__________________________________________________________________________________________________________________________________________________________________________________________________________________________________________

56. What do the sample sizes above refer to?

1. Crimes
2. People
3. Geographic areas
4. Other (specify)

56. Specify (other) ________________
57. Did the study report the number of stops or searches conducted?

1. Yes
2. No/unclear

57. If yes, how many stops were conducted for the treatment group? ________
58. If yes, how many searches were conducted for the treatment group? _______
59. If yes, how many stops were conducted for the control group? _________
60. If yes, how many searches were conducted for the control group? _________
61. Was demographic information provided for the people stopped/searched?

1. Yes
2. No

58. If yes, indicate the page numbers on which these data are provided ________
59. Did the study report the number of seizures (weapon or drug) that occurred?

1. Yes
2. No

59. If yes, how many weapon seizures occurred in the treatment group? __________
60. If yes, how many drug seizures occurred in the treatment group? ____________
61. If yes, how many weapon seizures occurred in the control group? ____________
62. If yes, how many drug seizures occurred in the control group? ______________

***Effect Size Data***

60. Raw difference favors (i.e. shows more success for):

1. Treatment group
2. Control group
3. Neither (exactly equal)
4. Cannot tell (or statistically insignificant report only)

61. Did a test of statistical significance indicate statistically significant differences between either the control and treatment groups or the pre and post tested treatment group for the current outcome? ____

1. Yes
2. No
3. N/A (no testing completed/reported)

61. If yes, what was the level of statistical significance for the current outcome

1. .01
2. .05
3. .1
4. N/A (no p‐value reported)

61. Type of significance test used for the current outcome

1. One‐tailed
2. Two‐tailed

9. Cannot tell (unclear from text or not reported)

62. Was an effect size reported?

1. Yes
2. No

62. What type of effect size was reported? _______________
63. If yes, what was the effect size? ______
64. If yes, page number where effect size data is found ________
65. If no, is there data available to calculate an effect size?

1. Yes
2. No

66. Type of data effect size can be calculated from (Select all that apply):

1. Means and standard deviations
2. *t*‐value or *F*‐value
3. Chi‐square (df=1)
4. Frequencies or proportions
5. Incident rate ratio, odds ratio, or risk ratio
6. Other (specify)

66. Specify (other) _________

*Pre‐post Study Counts*

67. Pre‐period number of events for current outcome in target area _______
68. During intervention‐period number of events for current outcome in target area ______
69. Post‐period number of events for current outcome in target area ______
70. Pre‐period number of events for current outcome in comparison area _______
71. During intervention‐period number of events for current outcome in comparison area _____
72. Post‐period number of events for current outcome in comparison area ______
73. Did the evaluation control for validity by using multivariate methods (i.e. regression) to assess the impact of the program?

1. Yes
2. No

67. If yes, did this analysis find that the intervention reduced the outcome at a statistically significant level?

1. Yes
2. No
3. N/A

*Means and Standard Deviations*

68. Pre‐period treatment group mean. _____
69. During intervention‐period treatment group mean_____
70. Post‐period treatment group mean_____
71. Pre‐period control group mean. _____
72. During intervention‐period control group mean_____
73. Post‐period control group mean_____
74. Pre‐period treatment group standard deviation. _____
75. During intervention‐period treatment group standard deviation. ____
76. Post‐period treatment group standard deviation. _____
77. Pre‐period control group standard deviation. _____
78. During intervention‐period control group standard deviation. _____
79. Post‐period control group standard deviation. ______

*Proportions or frequencies*

70. *n* of treatment group with a successful outcome. _____
71. *n* of control group with a successful outcome. _____
72. Proportion of treatment group with a successful outcome. _____
73. Proportion of control group with a successful outcome. _____

*Significance Tests*

72. *t*‐value _____
73. *F*‐value _____
74. Chi‐square value (*df*=1) _____
75. IRR value _______
76. OR value _______
77. RR (relative risk) value _______

*Calculated Effect Size*

73. Effect size ______
74. Standard error of effect size _____

**Conclusions made by the author(s)**

*Note that the following questions refer to conclusions about the effectiveness of the intervention. Desirable changes could be increases or decreases in the level of an outcome*

74. Conclusion about the impact of the intervention on crime/disorder? _____

1. Authors conclude intervention associated with crime decline
2. Authors conclude intervention not associated with a crime decline
3. Mixed across crime/disorder type
4. Unclear/no conclusion stated by authors

75. Conclusion about the impact of the intervention on violence in police/citizen encounters? _____

1. Authors conclude intervention associated with decline in violence
2. Authors conclude intervention not associated with decline in violence
3. Mixed evidence/no change
4. Unclear/no conclusion stated by authors

76. Conclusion about the impact of the intervention on officer misbehavior? ________

1. Authors conclude intervention associated with decline in misbehavior
2. Authors conclude intervention not associated with decline in misbehavior
3. Mixed evidence/no change
4. Unclear/no conclusion stated by authors

77. Conclusion about the impact of the intervention on community outcomes? ________

1. Authors conclude intervention associated with improvements in community outcomes
2. Authors conclude intervention not associated with improvement in community outcomes
3. Mixed evidence across outcome type
4. Unclear/no conclusion stated by authors

78. Did the assessment find evidence of a geographic displacement of crime? ______

1. Yes
2. No
3. No statistical test but authors claim evidence of displacement
4. No statistical test but authors claim no evidence of displacement
5. Mixed across crime/disorder type
6. Not tested

79. Did the assessment find evidence of other non‐geographic types of displacement of crime? _____

1. Yes
2. No
3. No statistical test but authors claim evidence of displacement
4. No statistical test but authors claim no evidence of displacement
5. Mixed across crime/disorder type
6. Not tested

79. If yes, specify what types of displacement were found

______________________________________________________________________________________________________________________________________________________

80. Additional notes about conclusions:

________________________________________________________________________________________________________________________________________________________

81. Additional notes about study:

__________________________________________________________________________________________________________________________________________________________
